# Molecular Mechanisms of Transfusion-Associated Immunomodulation and Its Impact in the Critically Ill

**DOI:** 10.3390/ijms27010030

**Published:** 2025-12-19

**Authors:** Angel Augusto Pérez-Calatayud, Klaus Görlinger

**Affiliations:** 1 Head of Critical Care Division, Hospital General de Mexico Dr Eduardo Liceaga, Mexico City 06720, Mexico; 2 Department of Anesthesiology and Intensive Care Medicine, University Hospital Essen, University Duisburg-Essen, 45147 Essen, Germany; kgoerlinger@werfen.com; 3 Tem Innovations, 81829 Munich, Germany

**Keywords:** TRIM, Transfusion, PBM

## Abstract

Allogeneic blood transfusion is frequently performed in critically ill patients, but accumulating evidence demonstrates that it is not a biologically neutral intervention. Transfusion-associated immunomodulation (TRIM) encompasses the immunological effects of transfusion, ranging from immune suppression to proinflammatory activation and cancer recurrence, with potential impact on morbidity and mortality in the intensive care unit. We conducted a narrative review of recent experimental and clinical evidence on TRIM to describe the molecular pathways involved. We reviewed, randomized trials, metaanalyses, and large observational cohorts to evaluate the clinical relevance of TRIM in critically ill populations. TRIM arises from multiple converging mechanisms. These pathways alter innate and adaptive immunity, leading to increased risk of healthcare-associated infections, transfusion-related acute lung injury, acute kidney injury, multiorgan dysfunction, prolonged length of stay, and cancer recurrence in surgical patients. Blood-sparing strategies, including patient blood management (PBM), mitigate exposure. The impact of storage duration and novel processing technologies remains unclear. There is still a gap in research that needs to be addressed. Transfusion-associated immunomodulation (TRIM) is a phenomenon in which donor leukocytes, extracellular vesicles, microparticles, bioactive lipids, and cytokines interact with the host immune system to produce a spectrum of immunological effects. In critically ill patients, the immune system is already fragile, and these mechanisms predispose patients to infections, pulmonary complications, organ dysfunction, prolonged recovery, and even cancer recurrence. Although TRIM cannot currently be diagnosed through a single biomarker or clinical test, its existence is strongly supported by mechanistic studies and consistent clinical associations between transfusion exposure and adverse outcomes.

## 1. Introduction

Allogeneic blood transfusion still is a common and overused treatment in modern critical care medicine, whose immunological consequences extend beyond simple oxygen delivery and coagulation management. Over the past three decades, transfusion-associated immunomodulation (TRIM) has emerged to describe transfusions’ complex and often paradoxical effects on the host immune system [1], causing a spectrum of immunological alterations that include proinflammatory responses and immunosuppressive effects, which influence patient outcomes with significant implications for infection risk, cancer progression, and organ dysfunction in critically ill patients [2,3]. The concept of transfusion-related immunomodulation, though formally named later, emerged from observations in the early 1970s regarding immunological changes in transfused patients [1]. These initial findings indicated that allogeneic blood exposure could induce an immunosuppressive effect, significantly improving renal allograft survival in transplant recipients [1]. This phenomenon, initially noted by Opelz et al., demonstrated that non-leukoreduced packed red blood cell transfusions provided a protective effect before the widespread availability of modern immunosuppressive agents [1,2]. Conversely, contemporaneous clinical observations in oncology revealed an increased incidence of postoperative infections and tumor recurrence in transfused patients, further supporting the multifaceted nature of allogeneic transfusion-related immunomodulation [3,4].

The molecular mechanisms of TRIM are multifactorial. It has been found to involve cellular and soluble mediators such as donor leukocytes, extracellular vesicles, microparticles, cytokines, bioactive lipids, and storage-related changes in transfused blood products. The interaction between the innate and adaptive immune responses induces a spectrum of immunomodulation that can mitigate or exacerbate disease processes in the critical care setting. Metabolomics has transformed our understanding of TRIM, emphasizing that transfusion is an active biochemical intervention rather than passive fluid replacement [3]. Critically ill patients often present systemic inflammation, immune dysregulation, and increased vulnerability to poor outcomes. With the given role of transfusion therapy, it is necessary to understand the biological underpinnings of TRIM.

This review summarizes the current evidence of the molecular mechanisms, their clinical relevance in critically ill patients, and strategies to mitigate their impact.

## 2. Methods

We conducted a narrative review of recent experimental and clinical evidence on TRIM to describe the molecular pathways involved. A structured literature search was performed in PubMed/MEDLINE, Embase, Web of Science, and the Cochrane Library for articles published between January 2013 and August 2025, using combinations of the following keywords and MeSH terms: “*transfusion-related immunomodulation*”, “*TRIM*”, “*blood transfusion*”, “*red blood cell storage*”, and “*extracellular vesicles*”. We included: (1) experimental and translational studies elucidating molecular or cellular mechanisms and (2) clinical studies (randomized controlled trials, meta-analyses, and large prospective or retrospective cohorts) reporting outcomes in adult or pediatric critically ill, surgical, trauma, septic, or oncologic populations. Case reports, small case series, conference abstracts without full text, and non-human studies without clear mechanistic relevance to human TRIM were not systematically considered.

## 3. Results

### 3.1. Molecular Mechanisms of Transfusion-Associated Immunomodulation

Outside alloantigens, transfusion products contain diverse immunomodulatory mediators capable of disrupting immune homeostasis. These factors can simultaneously trigger proinflammatory pathways, such as cytokine release and leukocyte activation, and exert immunosuppressive effects by impairing monocyte and T-cell function. The coexistence of these opposing responses may aggravate underlying inflammation, as seen in thromboinflammation during trauma-hemorrhagic shock [4]. The immunological consequences of transfusion are mediated through different overlapping molecular and cellular pathways categorized into effects related to cellular components, soluble mediators, and storage-related alterations of blood products. Together, they contribute to a complex immune dysregulation that can dampen host defense mechanisms, trigger inappropriate and potentially harmful inflammation, and activate coagulation pathways (Table 1).

Allogeneic leukocytes from residual donors are a critical driver of TRIM. The transfused allogenic leukocytes persist in the recipient, causing microchimerism and long-term immune tolerance. These changes suppress natural killer (NK) cell function, impair cytotoxic response, and expand regulatory T cells (Tregs) [5]. The increased risk of infections is caused by these changes, causing a reduced pathogen clearance, as a lasting impact of the transfusion [4,5]. Residual White Blood Cells (WBCs), particularly antigen-presenting cells (APCs) such as monocytes and dendritic cells, express major histocompatibility complex (MHC) class II molecules (e.g., HLA-DR) on their surfaces. Interactions between donor MHC class II molecules and recipient lymphocytes following transfusion drive alloimmunization or immune suppression by these molecules, which are critical for antigen processing and presentation to lymphocytes. The outcome is influenced by factors such as the degree of HLA compatibility, the functional status of donor APCs, and the inflammatory environment of the recipient [3,4,5].

The immune suppression seen in these scenarios is caused by the residual donor APCs that engage recipient T cells without providing the necessary secondary or costimulatory signals, resulting in antigen-specific T-cell anergy. This tolerance is a mechanism for transfusion-related adaptive immune suppression. The clinical manifestation is microchimerism, observed in the donor leukocytes that persist in the circulation of recipients who fail to mount an immune response against them [4]. Microchimerism has been documented particularly in trauma patients, in some cases persisting for up to two years post-transfusion. Such persistence may contribute to a shift toward immunosuppressive TH2-type responses observed in transfused patients [5]. There is limited direct causal evidence linking residual HLA-bearing APCs with post-transfusion immune suppression.

In addition to viable residual leukocytes, apoptotic donor WBCs in stored RBC units may also exert immunosuppressive effects. During storage, leukocytes may undergo apoptosis, with early phosphatidylserine exposure on the cell membrane as the key signal. The interaction between immune cells and phosphatidylserine promotes immunoregulatory pathways, further contributing to suppression in TRIM [4,5].

### 3.2. Hemolysis Byproducts

Free heme, iron, arginase, purine metabolites, and mitochondrial remnants represent a two-hit model. They can amplify inflammation through oxidative stress and innate immune activation and promote immune paralysis, pathogen growth, and tumor progression. Despite clear evidence that cell-free heme is released into the circulation after RBC breakdown, its precise concentration and how exactly it exerts its harmful effects remain unclear [6,7,8].

TRIM biology is complex and highlights the need for strategies to minimize hemolysis-driven immune perturbations in critically ill patients. Stored red blood cells undergo hemolysis, releasing hemoglobin, which degrades into free heme and iron. These two bioproducts, considered damage-associated molecular patterns (DAMPs), activate innate immune sensors and cause redox imbalance [7,9]. Free heme and iron catalyze the formation of reactive oxygen species via Fenton chemistry, leading to oxidative tissue injury and inflammation [8]. In animal models, transfusion of long-stored units is associated with surges in non-transferrin-bound iron and proinflammatory cytokines. Macrophage responses to heme and iron are complex and context-dependent [8,9,10,11]. On one hand, erythrophagocytosis and heme catabolism via heme oxygenase-1 (HO-1) promote an anti-inflammatory M2 phenotype, dampening inflammation through interleukin-10 (IL-10), biliverdin, and carbon monoxide signaling [10]. Conversely, iron loading impairs macrophage and T-cell function, reduces antigen presentation, and can trigger ferroptosis. Excess iron also inhibits lymphocyte proliferation and fuels bacterial and tumor growth, offering a potential explanation for the higher risk of sepsis and poor oncologic outcomes after transfusion [11,12].

Additional mediators also modulate immune function. The Arginase released during hemolysis depletes L-arginine, reducing nitric oxide bioavailability and impairing vasodilation and phagocyte function [13]. RBC-derived ATP and its metabolites (ADP, adenosine, hypoxanthine) exert immunomodulatory roles through purinergic signaling, with effects ranging from neutrophil activation to immunosuppression [13,14]. Donor genetic variability in enzymes such as CD38 or ARG1 influences the levels of these metabolites at the end of storage, adding further heterogeneity to transfusion outcomes [10]. Finally, residual mitochondria-containing reticulocytes and mitochondrial DNA fragments in some RBC units can act as immunostimulatory signals, recognized by the innate immune system as pathogen-associated molecular patterns due to their bacterial ancestry. These mitochondrial components may contribute to alloimmunization and inflammatory activation in transfusion recipients. Hemolysis byproducts, such as free heme, iron, arginase, purine metabolites, and mitochondrial remnants, represent a double-edged sword. In some situations, they amplify inflammation and promote immune suppression and pathogen growth [7,8,9,10,11,12,13].

Stored blood products accumulate bioactive cytokines and chemokines, including interleukin (IL)-10, IL-6, tumor necrosis factor-α (TNF-α), and transforming growth factor-β (TGF-β). These mediators modulate monocyte and T-cell activity, shifting the immune response and downregulating proinflammatory pathways [15,16,17,18]. Conversely, in the presence of a “first hit,” such as trauma or sepsis, these mediators may exacerbate systemic inflammation [19,20].

Erythrocyte and leukocyte-derived extracellular vesicles accumulate during storage and act as potent immunomodulators [21]. These vesicles deliver oxidized lipids, proteins, and nucleic acids to recipient immune cells, activating neutrophils and endothelial cells [22]. The result is enhanced oxidative stress, endothelial injury, and altered coagulation, all contributing to an inflamatory phenotype of this immune dysregulation observed in critically ill patients [23].

Bioactive Lipids and the Storage Lesion: lysophosphatidylcholines, a proinflammatory lipid, increase progressively during blood storage and have been implicated in neutrophil priming and pulmonary inflammation [24,25,26,27,28]. These “storage lesion” products amplify the risk of TRALI and have been shown to promote systemic immune activation and inflamatory response [29,30,31].

Antigen Presentation and HLA Molecules: When a recipient is exposed to a donor’s MHC, antigens enable immune tolerance by indirect antigen presentation pathways, inducing anergy in T cells, which promotes immunosuppression [32,33]. In oncology, these effects are linked to tumor recurrence and reduced immune surveillance [34,35,36,37]. TRIM represents the convergence of innate and adaptive immune alterations. The synergistic act of Neutrophil priming, monocyte/macrophage reprogramming, and T-cell modulation can generate a state of immune imbalance that predisposes patients to infection, delayed tissue healing, and, paradoxically, excessive inflammatory responses [33].

### 3.3. Pathophysiology of TRIM-Associated Complications in Critically Ill Patients

**Figure 1:**
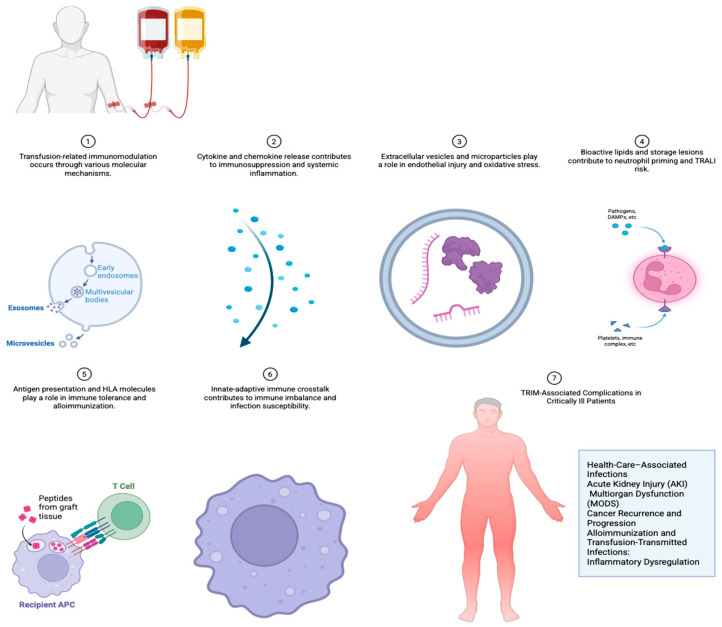
Molecular Mechanisms of TRIM and Their Clinical Consequences in Critically Ill Patients.

The adverse outcomes associated with transfusion in critical illness are associated with TRIM and have been consistently demonstrated with clinical data showing a correlation between transfusion and adverse outcomes [38]. Understanding the underlying pathophysiology provides a mechanistic framework to explain these associations [39,40] (Figure 1).

(A) Health-Care–Associated Infections (HCAI): Transfused allogeneic leukocytes and soluble mediators suppress innate and adaptive immune function, primarily by inhibiting natural killer (NK) cell cytotoxicity and expanding regulatory T cells (Tregs). Cytokines such as IL-10 and TGF-β, present in stored blood products, further downregulate proinflammatory pathways, impairing pathogen clearance. This state of relative immunosuppression predisposes critically ill patients, who are already vulnerable to systemic inflammation, to pneumonia, bloodstream infections, and sepsis [41,42,43,44,45].
(B) Pulmonary Complications: TRALI and Acute Lung Injury (ALI): The pathogenesis of TRALI is an example of the “two-hit” hypothesis [45]. In critically ill patients, the first hit is systemic inflammation caused by sepsis, trauma, or surgery, which primes pulmonary neutrophils and endothelial cells [46]. The second hit comes from transfused mediators such as anti-leukocyte antibodies, bioactive lipids, and extracellular vesicles, that activate primed neutrophils within the pulmonary microvasculature. This triggers capillary leak, oxidative damage, and an inflammatory cascade that culminates in acute lung injury or Acute Respiratory Distress Syndrome (ARDS) like syndromes [47,48,49,50,51,52].
(C) Acute Kidney Injury (AKI) and Multiorgan Dysfunction (MODS): Renal injury secondary to TRIM is caused by oxidative stress, alteration in the microcirculatory flow, and systemic inflammation [53,54]. Bioactive lipids and microparticles promote endothelial dysfunction, cause, disrupt renal perfusion, and contribute to tubular injury [55]. Additionally, cytokine imbalance propagates systemic immune dysregulation, extending the effect to multiple organs and amplifying the risk of MODS [56].
(D) Increased Length of Stay and Mortality: The combination of infection risk, pulmonary complications, and organ dysfunction directly translates into prolonged ICU and hospital stays [57]. Immunological derangements induced by transfusion impair the recovery from acute illness, and excessive inflammation contributes to complications. Although the impact on mortality remains inconsistent across trials, observational evidence has shown that cumulative transfusion correlates with worse survival, likely mediated by these pathophysiological pathways [58].
(E) Cancer Recurrence and Progression: In surgical oncology patients requiring intensive care, donor-derived leukocytes, HLA, and cytokines suppress cytotoxic T-cell and NK cell activity and promote regulatory pathways that favor tumor immune escape. This mechanism explains the association between perioperative transfusion and increased recurrence or metastasis in colorectal, urologic, and other solid tumors [34,37,59,60,61].
(F) Alloimmunization and Transfusion-Transmitted Infections: Alloimmunization occurs when the recipient’s immune system creates antibodies against foreign antigens from a donor, caused by the interaction between donor leukocytes and the recipient’s HLA [62]. Leukoreduction systems, which filter out white blood cells, effectively reduce this risk, but do not eliminate it because a small number of leukocytes and other foreign antigens can still be present [62]. Transfusion-transmitted infections (TTIs) can also occur, though leukoreduction has been shown to significantly decrease the incidence of TTIs like bacterial sepsis. Removing donor leukocytes and reducing the accumulation of cytokines during storage, leukoreduction decreases the potential for immune tolerance, alloimmunization, and infectious complications [63,64]. Although its impact on mortality has not been definitively proven, the biological plausibility and the consistent infection signals support its widespread use. Pre-storage leukoreduction is superior to post-storage approaches; timing of the intervention is critical [65,66,67,68]. Transfusion-transmitted cytomegalovirus (CMV) remains a concern in immunocompromised critically ill patients, as TRIM-related immunosuppression also enhances viral replication and impairs host defense [69,70,71,72,73].
(G) Inflammatory Dysregulation and the “Two Hit” Model: The most unifying feature of TRIM in the critically ill is its capacity to exacerbate preexisting inflammation [74]. In patients with trauma or sepsis, the immune system and its interaction with the endothelial cells are already activated; transfusion provides a secondary insult through extracellular vesicles, oxidized lipids, and cytokine accumulation [75]. The *two-hit model* explain how transfusion precipitates immune dysregulation and clinical complications. This model posits that an initial insult (1st hit) or pre-existing inflammatory state in the recipient primes the immune system, making it susceptible to a “second hit” from transfused blood products [74]. This secondary insult, (2nd Hit), mediated by biologically active components, exacerbate systemic inflammation or induce immunosuppression, leading to adverse clinical outcomes [74,75]. This dual-trigger mechanism reconciles the contradictory observations observed after transfusion, both immunosuppressive effects, and detrimental outcomes, such as increased infection rates and tumor recurrence [76].

The mechanisms of TRIM illustrate how transfusions act as a potent immunological modulator. TRIM’s interaction with both innate and adaptive immunity predisposes critically ill patients to infections, organ failure, pulmonary injury, tumor recurrence, and prolonged recovery [75,76].

It is necessary to understand that transfusion-associated immunomodulation (TRIM) has significant clinical implications for transfusion practice in critically ill patients, which endorses the need for transfusion stewardship and patient blood management (PBM) strategies to improve patient outcomes and minimize unnecessary transfusion and mitigate its immunomodulatory consequences [77,78,79]. Critically ill patients are vulnerable to these complications because of the presence of systemic inflammation, immune dysregulation, and multiorgan dysfunction, all of which are features of critical illness. Under these circumstances, the immunological effects of transfusion can exacerbate underlying pathophysiology and contribute to poor outcomes [26,57,76].

Implementing PBM programs is a more comprehensive approach. PBM is an evidence-based, systematic, patient-centered approach to improving patient outcomes [77]. It integrates preemptive anemia correction, minimization of blood loss and coagulopathy, and optimization of patients’ tolerance to anemia [77,79]. This multimodal bundle of care reduces exposure to transfusion and provides a structured and multidisciplinary strategy to mitigate the risks associated with TRIM.

The accumulated evidence from randomized controlled trials (RCTs) and metaanalyses has shown that restrictive transfusion strategies decrease infection rates and are at least as safe as liberal approaches [80,81,82,83,84,85,86,87]; however, PBM strategies completely mitigate this risk by managing the patients’ own blood [77,79,88,89,90,91,92,93,94,95]. These findings underscore the importance of limiting the negative consequences of TRIM by reducing unnecessary exposure through restrictive transfusion practices and PBM practices [77,78,79,80,81,82,83,84,85,86,87,88,89,90,91,92,93,94,95].

Also, blood storage age is linked to the “storage lesion,” characterized by a progressive accumulation of extracellular vesicles and bioactive lipids that can amplify TRIM [96,97]. However, the evidence from multicenter clinical trials failed to demonstrate a clear survival benefit of fresher red blood cells over standard-issue units in critically ill patients [98,99].

It is increasingly evident that not all patients are equally susceptible to TRIM. Sepsis, trauma, and major surgery patients are susceptible to the “two hit” phenomenon, where transfusion amplifies existing immune activation or suppression [13,100]. There is an urgent need to create risk-stratification tools and biomarkers to identify patients at the highest risk for TRIM complications and enable a more individualized treatment [65,76,98]. Emerging technologies, such as pathogen reduction, optimized storage methods, and extracellular vesicle depletion, are under active investigation and may further reduce the immunomodulatory effects of transfusion in the years to come [18].

TRIM is not defined as a discrete clinical syndrome with standardized diagnostic criteria [101,102]. TRIM is understood as a biological state resulting from immunological effects of allogeneic transfusion, predisposing patients to infection, organ dysfunction, and impaired immunomodulation. Because of this, diagnosis is inferential mainly, based on the temporal relationship between transfusion and subsequent adverse outcomes, supported by immunological or laboratory changes when available [44].

From a laboratory perspective, several immunological alterations have been described after transfusion: suppression of natural killer (NK) cell activity, expansion of regulatory T cells, polarization of monocytes and macrophages toward an anti-inflammatory phenotype, and shifts in cytokine balance, with elevated levels of IL-10 and transforming growth factor-β [103]. In some cases, microchimerism with persistence of donor leukocytes has been detected, reinforcing the concept of long-term immune modulation. Although these markers are not yet available for routine bedside practice, they provide mechanistic evidence of TRIM in experimental and research contexts [104].

## 4. Plasma, Platelet, and Cryoprecipitate Mechanisms of TRIM

TRIM has traditionally centered on red blood cell transfusions. However, cumulative evidence shows that plasma, platelet concentrates, and cryoprecipitate also have significant immunomodulatory effects [105,106,107,108,109,110,111]. These products contain a wide range of bioactive molecules, including cytokines, chemokines, lipid mediators, complement fragments, extracellular vesicles, microparticples, and soluble HLA antigens, that can profoundly influence immune homeostasis in the transfused recipient [1,2,105] (Table 2).

Fresh frozen plasma (FFP) contains soluble proteins for coagulation, complement activation, and immune regulation. Storage FFP accumulates bioactive lipids and inflammatory mediators, which affect endothelial permeability and leukocyte function. FPP also contains cytokines, such as IL-6, IL-8, IL-10, TNF-α, and TGF-β and the balance between pro- and anti-inflammatory cytokines vary depending on donor characteristics, pathogen reduction methods, and storage duration [3,4]. Another major pathway of plasma induce TRIM is complement activation. C3a and C5a can trigger leukocyte recruitment, mast cell degranulation, and endothelial activation, leading to systemic inflammation and increased vascular permeability. There is also a suppressed dendritic cell maturation and antigen presentation, which promotes immune tolerance. This exemplifies the paradoxical coexistence of immune activation and suppression within the same patient normally observed in TRIM [106,107]. Plasma transfusion has also been associated with the transfer of soluble HLA antigens, microparticles, and minor histocompatibility antigens capable of interacting with recipient lymphocytes. These interactions can modulate T-cell and NK-cell responses, occasionally leading to alloimmunization but more often producing transient immunosuppression. Moreover, storage-related oxidative stress generates oxidized phospholipids and lysophosphatidylcholines, priming neutrophils and monocytes for exaggerated inflammatory responses—a mechanism implicated in transfusion-related acute lung injury (TRALI) [107,108,109,110]

In trauma and massive transfusion scenarios, plasma may exert anti-inflammatory or immunorestorative effects when administered early, potentially through restoration of endothelial integrity and modulation of the glycocalyx [108,109]. This underscores the context dependent nature of TRIM: in some situations, plasma may stabilize immune function, while in others it may exacerbate inflammation or immune dysfunction. The challenge for clinicians and researchers is delineating which molecular signatures predict beneficial versus harmful immunomodulation [110].

Platelets are now understood as active participants in immune regulation. Platelet transfusions introduce a substantial immunological response that extends beyond clot formation. Stored platelet concentrates contain soluble CD40 ligand (sCD40L), platelet-derived (pEVs), microparticles, cytokines, chemokines (e.g., RANTES/CCL5), and arachidonic acid metabolites such as thromboxane A_2_ and Leukotriens [111]. These bioactive molecules are released progressively during storage and can modulate innate and adaptive immunity upon transfusion. sCD40L, one of the most potent immunomodulators released from activated platelets, binds to CD40 receptors on monocytes, macrophages, and dendritic cells. This interaction promotes upregulation of stimulary molecules, cytokine release, and antigen presentation [111]. In recipients with systemic inflammation, such activation can amplify cytokine storms and endothelial injury, contributing to TRALI and systemic inflammatory response syndrome (SIRS). Conversely, repeated exposure to platelet derived mediators may lead to immune exhaustion, T-cell dysfunction, and impaired microbial clearance, hallmarks of immunosuppression observed in chronically transfused or critically ill patients [112]. pEVs also represent key mediators of TRIM. These vesicles carry surface phosphatidylserine, CD41 (integrin αIIb), HLA class I molecules, and internal cargo including microRNAs, mitochondria, and immunomodulatory proteins. pEVs can activate neutrophils and monocytes, trigger coagulation cascades, and promote endothelial activation [111,112,113,114]. Notably, platelet concentrates with high EV content have been associated with an increased incidence of febrile non-hemolytic transfusion reactions and TRALI-like events [105,115,116]. Platelets interact directly with immune cells through toll-like receptors (TLRs) and Fcγ receptors, recognizing pathogen-associated molecular patterns and immune complexes. This crosstalk also potentiate both innate and adaptive responses. In the context of repeated or high-volume transfusion, the cumulative effect of platelet derived mediators can shift toward immune tolerance. This process involves the induction of regulatory T cells, suppression of dendritic cell function, and secretion of IL-10, thereby reproducing the immunosuppressive arm of TRIM [105,114]. Donor related variables also influence the immunological profile of platelet units. Donor age, sex, and inflammatory state affect baseline platelet activation and the release of mediators. Pathogen reduction technologies (e.g., amotosalen/UVA) can alter platelet membrane integrity and the release of EVs, potentially modifying TRIM-related outcomes.

Finally cryoprecipitate, though often viewed as a purely hemostatic product, also carries immunologically active proteins. It contains fibrinogen, von Willebrand factor (vWF), factor VIII, fibronectin, and vitronectin, having a role in immuneregulation [116]. These proteins can bind to integrins (e.g., αvβ3, α5β1) and toll-like receptors on immune and endothelial cells, influencing leukocyte adhesion, cytokine production, and angiogenesis [117]. Fibrinogen and vWF, in particular, have been shown to modulate macrophage polarization and dendritic cell function. Engagement of fibrinogen with macrophage integrins promotes a proinflammatory phenotype via NF-κB activation. In contrast, degradation products of fibrin (D-dimers, fragment E) can induce anti-inflammatory responses depending on the microenvironment. Thus, the net immune effect of cryoprecipitate likely depends on the recipient’s baseline inflammatory status and the dynamic balance between procoagulant and fibrinolytic processes [116,117]. Fibronectin and vitronectin, also act as DAMPs when exposed during tissue injury. They interact with toll-like and complement receptors, amplifying inflammatory cascades and enhancing phagocytosis. In surgical and trauma patients, these interactions may contribute to endothelial activation and secondary organ injury [116]. Furthermore, cryoprecipitate contains trace amounts of microparticles and EVs derived from plasma and platelets, which can further modulate immune responses in the transfused host [117].

## 5. Cell-Type–Specific Mechanisms Underlying TRIM

TRIM results from the interaction between host immune priming and transfusion-derived immune mediators. These trigger cell-type–specific molecular responses that alter innate and adaptive immunity [33].

Neutrophils: Primed neutrophils become activated through TLR4, TLR2, and NOD-like receptor (NLR) pathways in response to DAMPs and PAMPs, which are amplified in critical illness. Transfusion-derived extracellular vesicles (EVs), oxidized phospholipids, and lysophosphatidylcholines activate downstream NF-κB, MAPK, PI3K/AKT cascades, promoting degranulation and ROS production via NADPH oxidase (NOX2) [118,119,120]. NETosis is triggered by PAD4-mediated histone citrullination, leading to the extracellular release of neutrophil chromatin, which precipitates microvascular injury. Complement activation (C5a) further enhances neutrophil chemotaxis and endothelial adhesion, linking molecular danger signaling [121,122].

Monocytes and Macrophages: Monocytes respond to transfusion mediators via TLR4–MyD88, IL-10R–STAT3, and A20/TNFAIP3 regulatory pathways. This leads to a reduced HLA-DR/MHC-II expression and dampened antigen presentation [106,123]. Donor leukocytes shift monocyte metabolism to an immunosuppressive phenotype, characterized by increased oxidative phosphorylation, reduced glycolysis, and suppression of IRF5-dependent proinflammatory transcription [124]. This molecular polarization impairs phagocytosis, reduces TNF-α production, and promotes immune paralysis. Such immunosuppressive alterations can be further induced by erythrophagocytosis, where macrophages or monocytes internalize damaged red blood cells, leading to epigenetic and metabolic changes that impair their ability to respond to subsequent immune challenges, such as invading pathogens or inflammatory stimuli [1].

T Cells: Donor antigens and transfusion EVs exposure to T-cells modulates TCR signaling, resulting in reduced activation of ZAP-70, LAT, and NFATn [108]. Chronic alloantigen exposure induces inhibitory checkpoint molecules (PD-1, CTLA-4, TIM-3), thereby reducing IL-2 production and promoting T-cell anergy [125]. The expansion of regulatory T cells is driven by IL-10, TGF-β, and FoxP3-enhancing EVs, twisting immunity into a state of immune tolerance (tolerogenic state). These molecular pathways decrease tumor immune surveillance and impair pathogen clearance, linking transfusion events with postoperative infection and an increased risk of cancer recurrence [33,126,127].

B Cells: Transfusion-derived alloantibodies (HLA, HNA) and EVs modulate B-cell receptor (BCR) signaling via SYK, BLNK, and BTK [33,44,108,128]. This alters the memory of B-cell responses and antibody production. Donor immunoglobulins inhibit FcγR-mediated cytotoxicity and modulate complement activation (classical pathway, C1q–C4–C2), contributing to antibody-mediated TRALI. EV miRNAs (e.g., miR-150, miR-29b) influence B-cell differentiation and survival via BAFF/BLyS and APRIL pathways, impairing humoral immunity and predisposing to alloimmunization or delayed hemolytic transfusion reactions [24,98,129,130,131,132].

Platelets: Bioactive lipids accumulated during storage, especially, lysophosphatidylcholines and eicosanoid derivatives, bind platelet GPCRs (GPR40/120), to activate SRC family kinases, Syk, and PLCγ2, driving platelet activation [133,134,135,136]. Platelet-derived microparticles, rich in tissue factor (TF), promote coagulation and interact with neutrophils via P-selectin/PSGL-1, intensifying thromboinflammatory loops central to TRALI and acute lung injury. EVs modulate downstream RhoA/ROCK and integrin αIIbβ3 signaling, enhancing microthrombus formation and endothelial activation [137,138,139,140].

Endothelial Cells: Endothelial cells respond robustly to transfusion-derived cytokines (IL-1β, TNF-α), oxidized lipids, and alloantibodies through NF-κB, JAK/STAT, and p38 MAPK activation pathways. This induces upregulation of ICAM-1, VCAM-1, and E-selectin, facilitating leukocyte recruitment [135,136,137]. ROS production and mitochondrial dysfunction lead to cytoskeletal contraction via RhoA–MLC phosphorylation, increasing vascular permeability, a defining feature of TRALI. Complement deposition (C3a, C5a) further amplifies endothelial activation and promotes microvascular leak and injury [49,135,136,137,138,139,140,141,142].

Natural Killer Cells: After transfusion, NK cells display reduced activation of cytotoxic granule pathways, including decreased transcription of perforin and granzyme B through impaired STAT5 signaling [49,143,144,145]. Downregulation of activating receptors (NKG2D, NKp30, NKp46) and altered IL-12/IL-18 responsiveness suppresses NK-mediated tumor and pathogen clearance. These molecular defects contribute to increased infection susceptibility and may enable postoperative tumor progression [146].

Dendritic Cells: Dendritic cells (DCs) internalize transfusion EVs that contain immunomodulatory miRNAs, HLA molecules, and lipids, thereby reprogramming DCs toward a tolerogenic state [147]. This process involves enhanced PD-L1 expression, impaired CD80/CD86 co-stimulation, and suppression of IRF8 and NF-κB transcriptional programs required for effective antigen presentation, reduced processing of alloantigens and microbial antigens, weakened T-cell priming, and enhanced systemic immune tolerance [148,149].

These cellular molecular pathways show that TRIM is a coordinated network of cell-type–specific signaling events, integrating, pattern-recognition receptor activation (TLRs, NLRs), cytokine signaling (IL-10/STAT3, TNF-α/NF-κB, IL-2/STAT5), immune checkpoint induction (PD-1, CTLA-4), extracellular vesicle–mediated epigenetic modulation, complement activation (C5a-driven amplification loops), Immunometabolic reprogramming (shift toward OXPHOS or glycolysis suppression) and endothelial barrier disruption via RhoA/ROCK pathways. This molecular architecture provides a unified mechanistic explanation for the dual phenotype of TRIM hyperinflammation (e.g., TRALI) and immunosuppression (e.g., postoperative infections, tumor recurrence) in transfused patients.

## 6. Research Frontiers

Ongoing research in proteomics, metabolomics, and transcriptomics is being conducted to identify early molecular signatures of TRIM. Potential biomarkers include extracellular vesicles, oxidized lipids, and soluble HLA antigens, although none have been validated clinically. TRIM should be considered a syndrome of suspicion rather than a formal diagnosis. Its recognition relies on awareness of clinical patterns and exclusion of other causes of immune dysfunction [40,76]. TRIM has entered a new phase, with technological advances in immunology, product processing, and critical care research [117]. At the biological level, metabolomics and proteomics generate detailed maps of the biochemical and immunological networks activated after transfusion. Investigations are beginning to identify molecular signatures that could serve as future biomarkers to stratify patients at risk for TRIM-related complications [48,98,150,151].

Extracellular vesicles (EVs) play a role in propagating inflammatory signaling. EVs are increasingly regarded not only as mediators of TRIM but also as potential biomarkers of product quality and predictors of patient response [62,63,64]. Translational studies are needed to test whether EV-guided transfusion strategies or EV-modifying interventions can mitigate these effects [14,62,152,153].

On the product engineering side, several interventions are under investigation. Optimization of leukoreduction performed before storage may decrease immunosuppressive sequelae compared with post-storage filtration, though prospective data in critically ill patients are lacking [69,154,155,156]. Pathogen-reduction technologies already used for platelets and plasma are being extended to red blood cells; their capacity to attenuate TRIM by altering nucleic acid signaling or EV composition represents a promising hypothesis for future clinical trials [98,157,158,159]. Similarly, efforts to mitigate the storage lesion through improved additive solutions and modified storage conditions seek to limit the accumulation of oxidized lipids and vesicles, even though randomized clinical trials have not yet demonstrated an outcome benefit of fresher blood in ICU patients [14,97,160,161,162].

RCTs continue to reinforce the efficacy of restrictive hemoglobin thresholds with promising results in decreasing infection risk, which clinically is the primary manifestation of TRIM [58,85,163,164,165,166,167]. However, some patient subgroups require further investigation to balance oxygen delivery with immunological safety. The development of precision transfusion medicine, using biomarkers of immune function, extracellular vesicle load, or host inflammatory state, can potentially individualize transfusion decisions and directly address TRIM risk [14,33,98,168,169,170].

The PBM approach in critically ill patients needs further investigation, too. While this concept could completely avoid TRIM risk by avoiding transfusion, there is a lack of evidence inside the ICU [87,171,172,173].

Critical gaps remain: no validated bedside biomarkers identify TRIM, mechanistic interventions have yet to be tested against clinically relevant outcomes, and standardized definitions of TRIM-related endpoints are lacking. Future research should focus on multicenter trials to establish diagnostic criteria. Also, probable targeted interventions to mitigate the immunomodulatory effects of transfusion [88] need to be investigated. It is crucial to investigate alternative strategies in special populations [174].

## 7. Conclusions

TRIM is a phenomenon in which donor leukocytes, extracellular vesicles, microparticles, bioactive lipids, and cytokines interact with the host immune system to produce a spectrum of immunological effects. In critically ill patients, the immune system is already fragile; these mechanisms predispose the patients to infections, pulmonary complications, organ dysfunction, prolonged recovery, and even cancer recurrence. Although TRIM cannot currently be diagnosed through a single biomarker or clinical test, its existence is strongly supported by mechanistic studies and consistent clinical associations between transfusion exposure and adverse outcomes. Universal prestorage leukoreduction and a comprehensive PBM program reduce TRIM-related risks. Evidence for mortality benefit remains inconclusive. Future progress will depend on translational research bridging mechanistic insights with clinical practice.

## Figures and Tables

**Table 1:** Molecular Mechanisms of TRIM and Their Clinical Consequences in Critically Ill Patients.

Molecular Mechanism	Key Pathways	Clinical Consequences
Allogeneic leukocytes & microchimerism	Persistence of donor leukocytes, NK cell suppression, Treg expansion	Increased risk of infections, impaired immune surveillance
Cytokine & chemokine release	IL-6, IL-10, TNF-α, TGF-β accumulation during storage	Immunosuppression, systemic inflammation, “two-hit” amplification in sepsis/trauma
Extracellular vesicles & microparticles	Release of oxidized lipids, proteins, nucleic acids from stored cells	Endothelial injury, oxidative stress, altered coagulation, proinflammatory signaling
Bioactive lipids & storage lesion	Lysophosphatidylcholines, lipid peroxidation products	Neutrophil priming, TRALI risk, exaggerated inflammatory responses
Antigen presentation & HLA molecules	Indirect presentation of donor antigens, T-cell anergy	Immune tolerance, potential link with tumor recurrence, alloimmunization
Innate–adaptive immune crosstalk	Neutrophil priming, monocyte/macrophage reprogramming, T-cell modulation	Immune imbalance → infection susceptibility, delayed healing, organ dysfunction

TRIM: transfusion-associated immunomodulation; NK: natural killer cells; Treg: regulatory T cells; TRALI: transfusion-related acute lung injury.

**Table 2:** Blood Component–Derived Mechanisms Contributing to TRIM.

Blood Component	Key Mediators	Proposed Mechanisms	Clinical Consequences
Residual leukocytes (WBCs: monocytes, dendritic cells, lymphocytes, neutrophils)	MHC class II molecules (HLA-DR), alloantigens, cytokines	Antigen presentation leading to alloimmunization or T-cell anergy; microchimerism from donor cell persistence; apoptotic WBCs with phosphatidylserine exposure inducing immunosuppressive signaling	Alloimmunization, immune tolerance, microchimerism, TH2 shift, increased infection risk
Red blood cells (RBCs)	Hemolysis byproducts: free heme, iron, arginase, ATP, extracellular vesicles (EVs), residual mitochondria	Oxidative stress via ROS (Fenton chemistry); macrophage reprogramming (M1 vs. M2); ferroptosis and immune paralysis with iron overload; arginine depletion → reduced NO; ATP/adenosine signaling modulating innate/adaptive immunity; mitochondrial DNA as PAMPs	Inflammation, immune suppression, infections, sepsis, tumor growth, impaired vasodilation, alloimmunization
Platelets	Soluble CD40 ligand (sCD40L), platelet-derived EVs, microparticles, cytokines, chemokines, bioactive lipids	Immune cell activation (monocytes, T cells); endothelial activation; amplification of inflammatory cascades; delivery of procoagulant and proinflammatory EVs	Thrombosis, TRALI, systemic inflammation, immunosuppression in chronic transfusion
Plasma proteins	Soluble HLA molecules, microparticples, cytokines, anaphylatoxins (C3a, C5a), bioactive lipids	Modulation of innate/adaptive immunity; complement activation; induction of tolerance or inflammation depending on context	Infection risk, organ dysfunction, alloimmunization, anaphylactic reactions
Extracellular vesicles (from RBCs, platelets, WBCs)	MicroRNAs, proteins, oxidized phospholipids, mitochondrial fragments	Act as immune modulators by transferring bioactive molecules; neutrophil priming; endothelial activation; coagulation dysregulation	TRALI, systemic inflammation, microvascular injury, organ dysfunction
Storage lesion products (common to all cellular components)	Cytokines (IL-1, IL-6, IL-10, TNF-α), chemokines, oxidized lipids, free radicals	Accumulation during storage; trigger proinflammatory or immunosuppressive responses upon transfusion	Increased infection risk, inflammatory dysregulation, TRIM amplification

PC: Antigen-presenting cell, ATP: Adenosine triphosphate, CD40L: Cluster of differentiation 40 ligand, EVs: Extracellular vesicles, HO-1: Heme oxygenase-1, HLA: Human leukocyte antigen, IL: Interleukin, MHC: Major histocompatibility complex mtDNA: Mitochondrial DNA, NO: Nitric oxide, PAMPs: Pathogen-associated molecular patterns, RBCs: Red blood cells, ROS: Reactive oxygen species, sCD40L: Soluble CD40 ligand, TH2: T helper 2 cells, TRALI: Transfusion-related acute lung injury, TRIM: Transfusion-associated immunomodulation, WBCs: White blood cells.

## Data Availability

No new data were created or analyzed in this study. Data sharing is not applicable to this article.

## Abbreviations

APC	Antigen-presenting cell
ATP	Adenosine triphosphate
CD40L	Cluster of differentiation 40 ligand
EVs	Extracellular vesicles
HLA	Human leukocyte antigen
HO-1	Heme oxygenase-1
IL	Interleukin
MHC	Major histocompatibility complex
mtDNA	Mitochondrial DNA
NO	Nitric oxide
PAMPs	Pathogen-associated molecular patterns
RBCs	Red blood cells
ROS	Reactive oxygen species
sCD40L	Soluble CD40 ligand
TH2	T helper 2 cells
TRALI	Transfusion-related acute lung injury
TRIM	Transfusion-associated immunomodulation
WBCs	White blood cells
